# Obesity and Psychological Factors Associated with Weight Loss after Bariatric Surgery: A Longitudinal Study

**DOI:** 10.3390/nu14132690

**Published:** 2022-06-28

**Authors:** Serena Marchitelli, Eleonora Ricci, Cristina Mazza, Paolo Roma, Renata Tambelli, Giovanni Casella, Lucio Gnessi, Andrea Lenzi

**Affiliations:** 1 Department of Experimental Medicine, Sapienza University of Rome, 00161 Rome, Italy; serena.marchitelli@uniroma1.it (S.M.); lucio.gnessi@uniroma1.it (L.G.); andrea.lenzi@uniroma1.it (A.L.); 2 Department of Neuroscience, Imaging and Clinical Sciences, G. d’Annunzio University of Chieti-Pescara, 66100 Chieti, Italy; eleonora.ricci@unich.it; 3 Department of Human Neuroscience, Sapienza University of Rome, 00185 Rome, Italy; paolo.roma@uniroma1.it; 4 Department of Dynamic Clinical and Health Psychology, Sapienza University of Rome, 00185 Rome, Italy; renata.tambelli@uniroma1.it; 5 Department of Surgical Science, Sapienza University of Rome, 00161 Rome, Italy; giovanni.casella@uniroma1.it

**Keywords:** bariatric surgery, impulsiveness, alexithymia, binge eating, BMI change, weight loss

## Abstract

Bariatric surgery is indicated for treatment of severely obese people and can lead to significant weight loss as well as reduction of comorbidities associated with obesity. The present study aims to investigate the relationship between different psychological factors (e.g., tendency to binge eating, impulsivity, alexithymia), adherence to the nutritional plan, and weight loss after bariatric surgery. Forty-five candidates for bariatric surgery accessing a center for the care of obesity were assessed at T0 (pre-surgery) and T1 (6 months post-surgery) through anthropometric and psychometric measures. Simple linear correlations and linear regressions were conducted to evaluate the relationship between the psychological variables, adherence to nutritional plan, and weight loss 6 months after bariatric surgery. Non-planning impulsivity was the principal factor that succeeded in explaining adherence to the diet plan among all the variables considered. Adherence to the nutritional plan and non-planning impulsivity were considered reliable short-term predictors of weight loss after bariatric surgery. This evidence explains the usefulness of promoting research on psychological predictors of outcome in bariatric surgery. Mid- and long-term weight maintenance and quality of life need to be investigated through further follow-up.

## 1. Introduction

Obesity is a complex condition, which affects all ages and socioeconomic groups and carries serious psychological and social dimensions. Overweight and obesity pose a serious risk to individuals with diet-related chronic diseases, including type 2 diabetes, cardiovascular disease, hypertension, stroke, and some forms of cancer. Premature death and poor quality of life are consequences associated with obesity. Overweight and obesity are assessed by the body mass index (BMI), defined as the weight in kilograms divided by the square of the height in meters (kg/m^2^) [1]. An obese person has a BMI ˃ 30; obesity is divided into three categories: class 1 includes obese with BMI between 30 and 35; class 2 obese with BMI between 35 and 40; class 3 obese with BMI of 40 or higher. Class 3 is categorized as “severe” obesity [2]. The data of the Centers for Disease Control and Prevention of 2017–2018, compared with those of 1999–2000, report that in the last 20 years, the obese population in USA has increased from 30.5% to 42.4% [3]. 

Bariatric surgery is indicated for treatment of severely obese people and can lead to significant weight loss as well as reduction of comorbidities associated with obesity. The ideal candidate for bariatric surgery must have a class 3 obesity or class 2 but with comorbidities; tried all other weight loss methods; and agree to long-term follow-up after surgery. There are three types of bariatric surgery: gastric band, gastric bypass, and sleeve gastrectomy [4]. Bariatric surgery has been reported to have substantial and lasting effects on weight and to improve associated comorbidities; at the same time, surgery involves a degree of risk that is minimal in proportion to benefits [5]. Severe obesity is associated with a high prevalence of psychiatric conditions; psychological evaluation is an important factor before surgery, as these variables can affect the outcome [6]. A study investigated the literature on weight loss after bariatric surgery in patients with binge eating (BE), binge-eating disorder (BED), and loss of control eating (LOC); of the 15 studies considered, 14 reported lower loss and regain of lost weight in these categories [7]. Impulsivity is another factor often associated with negative surgical outcomes; a study reported that changes in behavioral measure of inhibitory control (SSRT) are positively correlated with positive surgical outcomes in terms of weight loss [8]. A study investigated the role of alexithymia in bariatric surgery candidates; it was found that patients with higher preoperative BMI and alexithymia lost a lower weight percentage (%EWL) than non-alexithymic patients [9].

Based on these assumptions, the present study aims to investigate the relationship between different psychological factors, the adherence to the nutritional plan, and weight loss in the short term (6 months after bariatric surgery).

## 2. Materials and Methods

### 2.1. Procedures

The project was launched from May 2018 to September 2021. All participants were recruited before bariatric surgery and underwent an anthropometric and psychological assessment. These anthropometric variables were height and weight (in order to estimate BMI). This procedure was repeated after 3 months, with the third at 6 months. Each participant gave informed consent, in which it was specified that the participant’s name and his or her answers would not appear anywhere, according to the European General Data Protection Regulation (GPDR; 2016/679 of The European Parliament and of The Council of 27 April 2016). All participants were briefed on the protocol and procedures. After administration, each patient was identified with a code to protect his identity. The experimental procedure was approved by the local ethics committee (Board of the Department of the Department of Experimental Medicine, Faculty of Medicine and Dentistry, Sapienza University of Rome), in accordance with the Declaration of Helsinki.

### 2.2. Participants

All participants were recruited at C.A.S.C.O (“Centro di Alta Specializzazione per la Cura dell’Obesità e delle malattie correlate”), a medical center specialized in treating obesity, at the University Hospital “Policlinico Umberto I” of Sapienza University, Rome. In the period between May 2018 and September 2021, 49 participants were recruited who requested bariatric surgery and passed the pre-surgical assessment (*N* = 49). Of the 49 participants, 45 accepted the protocol. Participants who underwent a gastric band were excluded from the research. The sample included 30 females and 15 males aged between 19 and 65 years.

### 2.3. Materials 

#### 2.3.1. Symptom Checklist-90-Revisited (SCL-90-R)

The Symptom Checklist-90—Revisited (SCL-90-R) [10] was adopted to estimate psychopathological symptoms. The scale is composed of 90 items, with a Likert scale ranging from 1 (not at all) and 5 (very much). The test represents 9 main psychological dimensions: somatization, obsessive-compulsive, interpersonal sensitivity, depression, anxiety, hostility, phobic anxiety, paranoid ideation, and psychoticism.

#### 2.3.2. Binge-Eating Scale (BES)

The Binge-Eating Scale (BES) [11] was adopted to evaluate the presence of episodes of binge eating in its behavioral food manifestation and in the feelings and cognitions that characterize the episodes. For each item, there are 3 or 4 possible answers, each with a specific score. The total score ranges from 0 and 46 points: if the total score is less than 17, the presence of symptoms of binge eating is unlikely; if it is between 17 and 27, it is possible; and if it is above 27, it is probable.

#### 2.3.3. Baratt Impulsiveness Scale-11 (BIS-11)

The Baratt Impulsiveness Scale-11 (BIS-11) [12,13] was adopted to measure impulsiveness. measure impulsiveness. The test allows to detect 3 components of impulsivity: attentive impulsivity, motor impulsivity, and non-planning impulsivity. The test consists of 30 items, with Likert scale ranging from 1 (rarely/never) and 4 (often/always).

#### 2.3.4. Toronto Alexithymia Scale-20 (TAS-20)

The Toronto Alexithymia Scale-20 (TAS-20) [14] was adopted to evaluate the construct of alexithymia; the questionnaire has 20 items, with a 5-point Likert scale ranging from 1 (strongly disagree) and 5 (strongly in agreement). Through this test, the three dimensions that define the construct of alexithymia are measured: difficulty in describing feelings (F1); difficulty identifying feelings (F2); and outward-oriented thinking (F3).

#### 2.3.5. Figure Rating Scale (FRS)

The Figure Rating Scale (FRS) [15] was adopted to determine body dissatisfaction in men and women. The test consists of 4 scales: 2 for women and 2 for men; each scale is made up of 9 silhouettes ranging from extreme thinness to extreme obesity. In the first scale, subjects are asked to indicate which silhouette their current figure corresponds to and, in the second, their ideal figure. Body dissatisfaction is evaluated based on the difference in score reported in the two scales.

#### 2.3.6. Obesity-Related Well-Being Questionnaire (ORWELL-97)

The Obesity-Related Well-Being Questionnaire (ORWELL-97) [16] was adopted to evaluate the quality of life (QoL) associated with obesity. The questionnaire has a 4-point Likert scale, ranging from 0 (for nothing) to 3 (very much). If the overall test score is ≥70, it can be considered indicative of a significant influence of obesity on the quality of life.

### 2.4. Statistical Analysis

Descriptive statistics of the pre-surgery sample on anthropometric and psychological variables were performed. Spearman’s correlation coefficients (rs) were calculated to study the associations between the psychological variables and the adherence to the prescribed nutritional plan (VAS, visual analytical scale) and the post-surgery weight loss expressed through BMI change, %EWL, and %TWL. The BMI change is the weight change between pre- and post-surgery (Δ BMI = pre-surgery BMI − post-surgery BMI); the %EWL corresponds to the excess weight loss (%EWL = (pre-surgery weight − post-surgery weight)/(pre-surgery weight − ideal weight) × 100) and the %TWL is the total weight loss (%TWL = (weight loss / pre-surgery weight) × 100). A linear regression analysis was used to determine the best predictors for the adherence to the prescribed nutritional plan (VAS). Using the stepwise method, the significant psychological variables were entered. Furthermore, two linear regressions were run, using the stepwise method, to determine the best predictors for the post-surgery weight loss and for both the post-surgery weight loss and the adherence to the prescribed nutritional plan (VAS). First, the BMI change was entered as a dependent variable and all the significant psychological variables as predictors. Then, in the second regression, the %TWL was considered as a dependent variable. Finally, no regression was carried out introducing %EWL as a dependent variable since no significant correlations were found between this index and the psychological variables considered and with the VAS. All statistical analyses were performed using SPSS Statistics 25.0 (IBM SPSS Statistics, New York, NY, USA).

## 3. Results 

### 3.1. Descriptive Statistics of the Sample

Table 1 summarizes the descriptive statistics of the anthropometric and psychological factors of the pre-surgery phase (T0). Both male and female participants were characterized by a tendency to somatization (*M* = 0.93, *SD* = 0.58, cut-off 1) and internalization (*M* = 0.85, *SD* = 0.62). Non-planning impulsivity averaged above the cut-off (cut-off = 2; *M* = 2.19, *SD* = 0.60). The tendency to binge-eating episodes averaged less than the cut off (cut-off = 17; *M* = 8.71, *SD* = 7.09) although with wide internal variability (range min = 0, max = 31).

### 3.2. Correlations

Table 2 summarizes the correlations between the psychological variables considered and (a) the adherence to the prescribed nutritional plan and (b) post-surgery weight loss expressed through BMI change, %EWL, and %TWL. Both BMI change and %TWL correlated significantly and negatively with BIS Total (rs_BMIchange_ = −0.422, *p* = 0.004; rs_%TWL_ = −0.525, *p* < 0.001) and with each subscale: BIS-NP (rs_BMIchange_ = −0.411, *p* = 0.005; rs_%TWL_ = −0.515, *p* < 0.001), BIS-M (rs_BMIchange_ = −0.390, *p* = 0.008; rs_%TWL_ = −0.480, *p* = 0.001), and BIS-C (rs_BMIchange_ = −0.312, *p* = 0.037; rs_%TWL_ = −0.364, *p* = 0.014). Furthermore, BMI change and %TWL correlated also significantly and negatively with both TAS Total (rs_BMIchange_ = −0.330, *p* = 0.027; rs_%TWL_ = −0.354, *p* = 0.017) and TAS-F1 subscale (rs_BMIchange_ = −0.318, *p* = 0.033; rs_%TWL_ = −0.363, *p* = 0.014). Both BMI change and %TWL correlated significantly and positively with the VAS (rs_BMIchange_ = 0.508, *p* < 0.001; rs_%TWL_ = 0.534, *p* < 0.001). Moreover, the VAS correlated significantly and negatively with SCL-90 O-C (rs_VAS_ = −0.336, *p* = 0.024), TAS-F1 (rs_VAS_ = −0.465, *p* = 0.001), TAS-F3 (rs_VAS_ = −0.321, *p* = 0.032), BIS Total (rs_VAS_ = −0.612, *p* < 0.001), BIS-NP (rs_VAS_ = −0.596, *p* < 0.001), BIS-M (rs_VAS_ = −0.496, *p* = 0.001) and BIS-C (rs_VAS_ = −0.564, *p* < 0.001), BES (rs_VAS_ = −0.413, *p* = 0.005), and ORWELL-97 (rs_VAS_ = −0.330, *p* = 0.027). No significant correlations were reported for the %EWL.

### 3.3. Regressions

Three multiple linear regressions were performed using the stepwise method (see Table 3). For the VAS, set as dependent variable, the BIS Total, the BIS-NP, the BIS-C, the BIS-M, the TAS Total, the TAS-F1, the SCL-90 O-C, the BES and the ORWELL-97 were considered as predictors. The multiple linear regression using the stepwise method identified as a significant predictor only the BIS-NP subscale (R^2^ = 0.356, (Adjusted R^2^ = 0.341), F-change (1,43) = 23.748, *p* < 0.001). The BIS Total scale and the TAS Total scale were excluded from the model for collinearity issues (VIF_BIS-TOT_ = 1664.772; VIF_TAS-TOT_ = 40.842). Considering the VAS as the dependent variable, with a number of predictors of *N* = 8, an observed R^2^ of 0.356, a significance level of 0.05, and a number of subjects of *N* = 45, the observed statistical power was 0.91.

Considering %TWL as a dependent variable, the BIS Total, the BIS-NP, the BIS-C, the BIS-M, the TAS Total, the TAS-F1, and the VAS were entered as predictors. The BIS Total scale was excluded from the model for collinearity issues (VIF_BIS-TOT_ = 1422.11). Using the stepwise method, only the BIS-NP subscale and the VAS scale (R^2^ = 0.394, (Adjusted R^2^ = 0.365), F-change (2,42) = 13.642, *p* < 0.001) were identified. For %TWL as dependent variable, with a number of predictors of *N* = 6, an observed R^2^ of 0.394, a significance level of 0.05, and a number of subjects of *N* = 45, the observed statistical power was 0.97.

Lastly, the BMI change was entered as a dependent variable and the BIS Total, the BIS-NP, the BIS-C, the BIS-M, the TAS Total, the TAS-F1, and the VAS were considered as predictors. The BIS Total scale was excluded from the regression for collinearity issues (VIF_BIS-TOT_ = 1415.59). Using the stepwise method, only the BIS-NP subscale (R^2^ = 0.254, (Adjusted R^2^ = 0.237), F-change (1,43) = 14.678, *p* < 0.001) was predictive of postoperative weight loss expressed as BMI change. Considering the BMI change as the dependent variable, with a number of predictors of *N* = 6, an observed R^2^ of 0.254, a significance level of 0.05, and a number of subjects of *N* = 45, the observed statistical power was 0.78.

## 4. Discussion

The present study sought to investigate the relationship between different psychological factors (i.e., psychopathological symptoms, tendency to episodes of binge eating, impulsiveness, alexithymia, body dissatisfaction, quality of life), the adherence to nutritional regimen, and the post-surgery weight loss in a sample of obese patients 6 months after a sleeve gastrectomy. Taking into account that 6 months is a short-term follow-up, it is considered as a first outcome; meanwhile, future follow-ups are nowadays already scheduled. 

The VAS (Visual Analytical Scale) correlated significantly, highly, and negatively with the impulsivity total scale (BIS 11-TOT) and correlated significantly, moderately, and negatively with each of the BIS subscales (BIS-NP, BIS-C, and BIS-M), highlighting how the greater the tendency to impulsivity, the lower the adherence to the nutritional regime prescribed. This result is in line with those of Tambelli et al. [17], Giel et al. [18], and Sarwer et al. [19]: impulsivity seems to contribute to the maintenance of obesity through an excessive and uncontrolled intake of food, maybe due to a difficulty in inhibition of dominant and intrusive behaviors and thoughts. A significant, negative, and low correlation was found also between the VAS and the quality of life (QoL; ORWELL-97). Specifically, the lower the self-perceived quality of life in the pre-surgery phase, the worse the adherence to the dietary regimen at six months after the procedure (high values at the ORWELL-97 subscale indicate low quality of life). The literature in this regard is contradictory. Studies on obesity-related quality of life mainly focused on improving it following bariatric procedures, but few studies investigated the link between preoperative quality of life and postoperative weight loss [20]. In general, subjects with higher BMI presented a worse quality of life and a more compromised psychopathological picture, with a greater tendency to impulsiveness. Some studies suggested also that the perception of good social and relational functioning (QoL) could play a moderating role of impulsiveness [21,22,23]. In our case, on the contrary, a low self-perceived quality of life could lead to poor adherence precisely because of the intervention of impulsiveness. The VAS correlated negatively and poorly with the O-C subscale of the SCL-90-R. This association highlights how subjects with obsessive-compulsive symptomatology have greater difficulty in observing the prescribed dietary regimen. Symptomatology related to obsessive-compulsiveness focuses on persistent thoughts and actions that are ego-dystonic or unwanted in nature [24]. The symptoms that are most investigated in the scale are related to difficulties in making decisions, concentrating, chasing away unwanted thoughts, and the tendency to act compulsively. All these characteristics were found in the literature to be strongly related to impulsiveness. Indeed, this kind of patient tends to prefer immediate rewards although they may lead to future negative consequences, has poor self-control in impulses—so much that they tend to compulsion—, and often presents risk behaviors, such as pathological gambling and substance dependence [25]. The fact that this symptomatology, characterized by the inability to control one’s unwanted thoughts or actions, is strongly linked to impulsiveness could explain why these patients are less adherent to dieting. Strongly impulsive traits could compromise patients’ ability to consider long-term consequences and make them lean towards seeking more immediate gratification [18,25]. Furthermore, a negative and moderate correlation between the VAS and the tendency to binge episodes was found. This result is of immediate and intuitive interpretation: the greater the loss of control related to nutrition expressed in terms of episodes of Binge, the lower the adherence to the post-surgery dietary plan. This finding is consistent with those of Kulendran et al. [8], who found that the presence of BED is a predictive factor of low weight loss after bariatric surgery. Finally, no significant correlations were reported between %EWL and psychological variables or VAS, indicating that other indices in addition to %EWL should be used in studies of subjects with obesity.

The most important evidence that emerged concerns the central role played by the impulsiveness in influencing both the adherence to the nutritional regimen (VAS) and the post-surgery weight loss. Using linear regression, it was possible to demonstrate the predictive role of impulsiveness (specifically the subscale of non-planning impulsiveness) on adherence to the nutritional plan. Non-planning impulsiveness is defined as the inability to predict the consequences of one’s actions and to plan them, inhibiting those unsuitable to achieve the goal [26]. It is likely, therefore, that this dimension of personality interferes with adherence, generating dysfunctional behaviors to weight loss [19,27]. Indeed, it was possible to demonstrate that both impulsiveness from non-planning and the adherence to the nutritional plan are reliable predictors of post-surgical weight loss (%TWL) at 6 months after the procedure. This result is within open debate in the literature: several studies identify impulsiveness as a central factor in determining post bariatric weight loss, while others highlight the central role played by other variables. Yeo et al. [28] performed a systematic review investigating the role of impulsiveness on post bariatric weight loss: they analyzed 10 studies with a total of 1246 patients with a follow-up ranging from 0.5 to 12 years. Of these, six studies showed no association between postoperative weight loss and impulsiveness, two studies reported an indirect effect mediated by eating disorders, and the remainder showed direct associations between the variables. This review clearly demonstrates the rift that exists in the literature on this subject and the need to investigate the field further.

Finally, alexithymia total scale (TAS TOT) and its subscales F1 (“difficulty identifying emotions”) and F3 (“outward-oriented thinking”) also appear to be associated with adherence to the post-surgery nutritional plan (negative correlation). This finding is in line with literature data that place alexithymia as a central factor in adherence to weight loss plans prescribed to surgical and non-surgical individuals with obesity [9,29]. However, alexithymia appears to have no predictive role on adherence leaving, i.e., the role of predictor to non-planning impulsiveness.

Overall, in our sample, impulsive and alexithymic subjects reported less weight change at 6 months after the bariatric procedure. Furthermore, the more the subjects adhered to the nutritional plan, the more they lost weight at 6 months after the bariatric procedure. Impulsiveness from non-planning and adherence to the dietary regimen were found to be reliable predictors of post-surgery weight loss (%TWL) at 6 months after the procedure.

The present study took into consideration a large set of variables, such as impulsivity, binge episodes, adherence, alexithymia, and psychopathology. This choice represents a novelty in the field of the longitudinal studies on weight loss after bariatric surgery. Only few psychological factors (one or two on average) associated to weight loss are usually considered in literature, and rarely is their association estimated. Moreover, more than one index has been considered for the evaluation of post bariatric weight loss (%EWL, %TWL, BMI change), which gives us a more accurate and reliable measure of it. Nevertheless, some limitations must be mentioned. First, the sample size, which allows a cautious generalization of the results, did not allow performing a separate analysis by gender. Another limitation concerns the brevity of the follow-up, carried out at 6 months. Further follow-ups are in progress to evaluate the role of the variables considered in the medium/long term.

## Figures and Tables

**Table 1:** Descriptive statistics of the anthropometric and psychological factors at T0 (pre-surgery phase).

Factors	Groups	*M*	*SD*
**Anthropometric Variables**	Weight	117.76	18.16
	BMI	41.91	4.22
	Age	43.73	13.73
**SCL-90-R** **Psychopathological Symptoms**	SCL-90 SOM	0.93	0.58
	SCL-90 O-C	0.67	0.47
	SCL-90 INT	0.85	0.62
	SCL-90 DEP	0.76	0.52
	SCL-90 ANX	0.46	0.40
	SCL-90 HOS	0.43	0.46
	SCL-90 PHO	0.25	0.35
	SCL-90 PAR	0.75	0.65
	SCL-90 PSY	0.25	0.43
	SCL-90 SLEEP	0.75	0.58
	SCL-90 GSI	0.63	0.38
**BIS-11** **Impulsivity**	Attentional Impulsivity	1.56	0.39
	Non-planning Impulsivity	2.19	0.60
	Motor Impulsivity	1.69	0.51
	Impulsivity Total	1.82	0.47
**TAS-20** **Alexithymia**	Alexithymia F1	15.53	5.72
	Alexithymia F2	10.11	4.68
	Alexithymia F3	13.56	6.93
	Alexithymia Total	39.20	14.74
**BES** **Binge-Eating Episodes**		8.71	7.09
**FRS** **Body Dissatisfaction**		−3.58	1.03
**ORWELL-97** **Quality of Life**		42.02	20.24

Note. Means (M) and standard deviations (SD) for all the anthropometric and psychological factors are reported in the third and fourth columns. SCL-90-R, Symptom Checklist-90—Revisited; BIS-11, Baratt Impulsiveness Scale-11; TAS-20, Toronto Alexithymia Scale-20; BES, Binge-Eating Scale; FRS, Figure Rating Scale; ORWELL-97, Obesity-Related Well-Being Questionnaire; BMI, body mass index (kg/m^2^), weight (kg).

**Table 2:** Correlations between the psychological variables and the BMI change, %EWL, and %TWL and the VAS.

	BMI-Change	%TWL	%EWL	VAS
**SCL-90 O-C**	−0.097	−0.110	−0.125	**−0.336**
*p*	NS	NS	NS	**0.024**
**BIS TOTAL**	**−0.422**	**−0.525**	−0.127	**−0.612**
*p*	**0.004**	**<0.001**	NS	**<0.001**
**BIS-NP**	**−0.411**	**−0.515**	−0.090	**−0.596**
*p*	**0.005**	**<0.001**	NS	**<0.001**
**BIS-C**	**−0.312**	**−0.364**	−0.001	**−0.564**
*p*	**0.037**	**0.014**	NS	**<0.001**
**BIS-M**	**−0.390**	**−0.480**	−0.106	**−0.496**
*p*	**0.008**	**0.001**	NS	**0.001**
**TAS-F1**	**−0.318**	**−0.363**	−0.077	**−0.465**
*p*	**0.033**	**0.014**	NS	**0.001**
**TAS TOTAL**	**−0.330**	**−0.354**	0.018	**−0.501**
*p*	**0.027**	**0.017**	NS	**<0.001**
**BES**	−0.156	−0.149	0.040	**−0.413**
*p*	NS	NS	NS	**0.005**
**ORWELL-97**	−0.169	−0.170	0.095	**−0.330**
*p*	NS	NS	NS	**0.027**
**VAS**	**0.508**	**0.534**	−0.025	
*p*	**<0.001**	**<0.001**	NS	

Note. Spearman’s correlation coefficients (rs) of the association between psychological variables and VAS, BMI change, %TWL, and %EWL are reported. BMI change, weight change; %TWL, total weight loss; %EWL, excess weight loss; VAS, visual analytical scale—adherence to the nutritional plan; SCL-90 O-C, Symptom Checklist-90, Obsessive-Compulsive; BIS-11 TOTAL, Baratt Impulsiveness Scale-11; BIS-11 NP, non-planning impulsivity; BIS-11 C, attentive impulsivity; BIS-11 M, motor impulsivity; TAS-20 F1, alexithymia F1—difficulty in describing feelings; TAS-20 TOTAL, Toronto Alexithymia Scale-20; BES, Binge-Eating Scale; ORWELL-97, Obesity-Related Well-Being Questionnaire. Statistically significant effects (*p* < 0.05) are in bold.

**Table 3:** Multiple linear regressions predicting VAS, %TWL, and BMI change.

		VAS	
	*B*	*t*	*p*
TAS-F1	−0.225	−1.495	NS
TAS-F3	−0.001	−0.007	NS
**BIS-NP**	**−0.596**	**−4.873**	**<0.001**
BIS-M	−0.310	−1.480	NS
BIS-C	−0.002	−0.012	NS
SCL-90 O-C	−0.132	−1.036	NS
BES	0.099	0.652	NS
ORWELL	−0.015	−0.119	NS
		**%TWL**	
	***B***	***t***	***p***
TAS-F1	0.007	0.043	NS
TAS-Total	0.022	0.146	NS
**BIS-NP**	**−0.340**	**−2.274**	**0.028**
BIS-M	−0.169	−0.786	NS
BIS-C	0.017	0.100	NS
VAS	0.362	2.417	0.020
		**BMI-change**	
	***B***	***t***	***p***
TAS-F1	−0.103	−0.622	NS
TAS-Total	−0.063	−0.392	NS
**BIS-NP**	**−0.504**	**−3.831**	**<0.001**
BIS-M	−0.212	−0.929	NS
BIS-C	0.017	0.090	NS
VAS	0.314	1.979	NS

Note. Coefficients from the multiple linear regressions (B and t) predicting VAS, %TWL, and BMI change and statistical significance (*p*) are reported in the three columns. VAS, visual analytical scale—adherence to the nutritional plan; %TWL, total weight loss; BMI change, weight change; TAS-20 TOTAL, Toronto Alexithymia Scale-20; TAS-20 F1, alexithymia F1—difficulty in describing feelings; TAS-20 F3, alexithymia F3—outward-oriented thinking; BIS-11 NP, non-planning impulsivity; BIS-11 M, motor impulsivity; BIS-11 C, attentive impulsivity; SCL-90 O-C, Symptom Checklist-90 Obsessive-Compulsive; BES, Binge-Eating Scale; ORWELL-97, Obesity-Related Well-Being Questionnaire. Statistically significant effects (*p* < 0.05) are in bold.

## Data Availability

The data that support the findings of this study are available from the corresponding author, C.M., upon reasonable request.
